# Regional Hypertrophy: The Effect of Exercises at Long and Short Muscle Lengths in Recreationally Trained Women

**DOI:** 10.5114/jhk/163561

**Published:** 2023-07-15

**Authors:** Aitor Zabaleta-Korta, Eneko Fernández-Peña, Jon Torres-Unda, Maider Francés, Asier Zubillaga, Jordan Santos-Concejero

**Affiliations:** 1 Sports and Education Department, University of the Basque Country (UPV/EHU), Vitoria-Gasteiz, Spain.; 2 Physiotherapy Department, University of the Basque Country (UPV/EHU), Leioa, Spain.

**Keywords:** muscle growth, selective hypertrophy, strength, variable resistance, muscle architecture

## Abstract

The aim of the present study was to analyse the role of exercises' resistance profile in regional hypertrophy. Thirty-eight healthy women completed a 9-week resistance training program consisting of either 4 sets of 12 repetitions to volitional failure of inclined bicep curls (INC group) or preacher curls (PREA group), three times per week. Pre- and post-intervention muscle thickness was measured using B-mode ultrasound imaging with a linear-array transducer. Scan acquisition sites were determined by measuring 50%, 60% and 70% of the distance between the posterior crest of the acromion and the olecranon. Statistical significance was set at p < 0.05. No region of the INC group grew when comparing pre- to post-intervention. The 70% region of the PREA group grew significantly (muscle thickness increased from 2.7 ± 0.43 cm to 2.94 ± 0.44 cm). We found no growth differences between regions when analysing per group (p = 0.274), region (p = 0.571) or group*region (p = 0.367). Our results show that the distal region of the arm grows in response to the preacher curl that places the highest amount of strain in the range of motion in which the arm muscles are more elongated.

## Introduction

Muscle growth, also known as muscle hypertrophy, is a complex process highly dependent on mechanical force (Schoenfeld et al., 2021; Wackerhage et al., 2019). It is related to performance improvements in many sports (Storey and Smith, 2012), and since a high muscle mass implies a lower mortality risk in different populations, it is important for health professionals to know how to prescribe exercise appropriately (Sedlmeier et al., 2021; Srikanthan et al., 2016; Srikanthan and Karlamangla, 2014; Wannamethee et al., 2007).

Muscle does not grow homogeneously (Franchi et al., 2018c; Matta et al., 2011; Narici et al., 1989; Nosaka and Sakamoto, 2000) as it can differ between the heads of a muscle and even between the different regions of a single head (Zabaleta-Korta et al., 2020). This phenomenon, known as regional hypertrophy, has drawn a considerable amount of attention in recent years (Diniz et al., 2020; Maeo et al., 2021; Martins-Costa et al., 2021). Several factors seem to influence regional hypertrophy, including exercise selection, a different number of repetitions with a normalized time under tension (Martins-Costa et al., 2021; Wilk et al., 2021), time spent in the concentric and eccentric phases of an exercise (Diniz et al., 2020), training at different muscle lengths (Maeo et al., 2021) or even performing only concentric/eccentric exercises (Franchi et al., 2014, 2018c). In all those studies, applied muscle force appears to be an important factor that somehow shapes which region of muscle will grow.

Recently, Zabaleta-Korta et al. (2021) found that exercise selection might elicit different regional hypertrophy stimuli. In that study, authors tested whether regional hypertrophy found after performing leg extensions was different to the one elicited by squats performed on the Smith machine in young men. For that purpose, they divided subjects into two groups and each group performed one exercise. After the intervention, hypertrophy of two regions in two different muscle heads was compared between and within regions. The authors found that exercise selection had an influence on regional hypertrophy, as it was different even if both groups had followed the same volume and intensity protocol. However, since the exercises studied had different resistance profiles (meaning they placed strain in different parts of the range of motion (RoM)), it was not possible to know whether the regional hypertrophy reported was due to the exercise selection or because the maximum resistance that the involved joints had to overcome occurred at different muscle lengths. Despite this limitation, those findings suggest that regarding the leg extension, the fact that the hip joint does not move while the knee extends may elicit more stimuli for the rectus femoris muscle. For the Smith machine squats, it can be inferred from those results that the vastus lateralis gets more growth stimuli in exercises that are similar to squats.

Comparisons between exercises with different resistance profiles such as bicep preacher curls and inclined bicep curls, defined as the degree of resistance in each part of the RoM of a joint, have been reported previously (Maeo et al., 2021; Wakahara et al., 2013), but not with the specific aim of reporting the effects of resistance profiles on regional hypertrophy. This may have confounding effects on our interpretation of the results and thus, the aim of the present study was to analyze the role of exercises with different resistance profiles in regional hypertrophy. For that purpose, inclined bicep curl and preacher bicep curl exercises were compared in three different regions of the arm. The first exercise places the highest amount of strain (represented as the greatest difficulty of the exercise) at the end of the exercise (Loss and Candotti, 2008), when the arm is at 90–100º flexion, while the latest places the highest amount of strain at the beginning (Loss and Candotti, 2008), with a considerable amount of elongation in the biceps brachii and brachialis muscles. In addition, we decided to perform the study in recreationally trained women, as most regional hypertrophy studies have analyzed this phenomenon in men. We hypothesized that the preacher curl would elicit greater muscle growth in the distal part of the biceps, as reported by previous research in which the highest strain occurred when the muscle was elongated (Nosaka and Sakamoto, 2000).

## Methods

### Participants

Thirty-eight healthy women joined this study. Participants were required to meet the following inclusion criteria: 1) women with an age ranging between 18 and 45 years; 2) lack of musculoskeletal disorders in the upper limbs at least one year prior to the beginning of the study, and 3) a resistance training experience of at least 6 months prior to the beginning of the study.

A total of 31 participants finished the study. Two participants dropped out because they tested positive for SARS-COVID19 and five participants dropped out for personal reasons not related to the study. Written informed consent was obtained from each participant after a thorough explanation of the testing protocol, the possible risks involved and the right to terminate participation at will was provided. The study was approved by the Institutional Review Board of the University of the Basque Country UPV/EHU (ref. 118/2019) and all procedures were conducted in accordance with the Declaration of Helsinki (2013). Participants were analyzed by the intention-to-treat principle in order to avoid the risk of bias (McCoy, 2017) even if two participants did not reach 90% of attendance to the planned training sessions. A priori sample size calculations were carried out according to the AIPE approach (Abt et al., 2020) performed with the ESCI software (Abt et al., 2020). This analysis revealed that to obtain a 95% confidence interval with a standardized mean difference of 0.4 between groups (Abt et al., 2020) and a width of 0.8 (0.4 in each side of the point estimate), each group should include at least 49 participants. Unfortunately, we were unable to reach such a high number of participants, thus the risk of a type II statistical error is high.

### Measures

Measurements were performed two days prior to the beginning of the training program and 72–96 h after its end. Measurements lasted ~30 min and testing sessions were carefully scheduled to ensure that the same number of hours were left since the end of the training protocol. During the day of the first measurements, participants were randomly allocated into the INC and PREA groups, using the RANDOM function of an Excel 2016 spreadsheet.

### Training Program

The two experimental groups trained both arms, despite the fact that only one arm was analyzed. This was made to avoid any potential cross-education effect (Lee and Carroll, 2007). Participants in the INC group completed a 9-week resistance training program consisting of 4 sets of 12 repetitions of inclined bicep curls three times per week, performing each set to muscular failure. Inclined bicep curls consisted of performing bicep curls with a dumbbell while lying supine on a bench with 45º inclination. The exercise began with the forearm perpendicular to the floor, and ended when the elbow reached at least a 90º angle, never going beyond 110º. Participants in the PREA group completed the same training protocol, but performed the preacher bicep curl instead of the inclined bicep curl. The preacher bicep curl consisted of performing bicep curls with a dumbbell on a Scott bench. A Scott bench is a specific kind of a bench consisting of a 50º inclined platform where the participant places the arm while performing the curling movement. The exercise began with the forearm parallel to the floor or slightly below, and ended when the forearm formed a 75º angle with the floor, never reaching a position perpendicular to the floor. Participants in both groups were requested to keep the hand always completely supinated to avoid the potential impact that the forearm position has on the recruitment of elbow flexors (Kleiber et al., 2015). They were also requested not to perform any other biceps brachii or pulling exercises (also known as back exercises), that would directly or indirectly affect the stimulus provided by the exercises of the study. To make it easier for participants to organize their training sessions with the conditions of the study, they were offered different training plans according to the number of days they wanted to train. They were also encouraged not to perform strenuous pulling or bicep-demanding activities during the period of the study.

Weekly training volume consisted of 12 weekly sets (Baz-Valle et al., 2018) for the biceps brachii exercise and varied between 60 and 85 sets for the rest of the muscles (depending on the number of days that each subject trained). This amount of weekly sets follows current guidelines of weekly resistance exercise volume to maximize muscle growth (Baz-Valle et al., 2022).

Training was programmed in a flexible fashion: on the first day, participants were told to adjust the weight so that they could reach muscular failure at 12 repetitions. For that purpose, participants were told to choose a weight that would make them reach, but not exceed 12 repetitions on their exercise. If they were able to perform 1–3 extra repetitions with the chosen weight, they had to use the next heaviest dumbbell. If the weight difference between dumbbells was too high (in some gyms, the weight difference between one dumbbell and the next heaviest one is 2.5 kg, and as most participants lifted 10 kg, that would be a 25% increase), participants were given two options: they could lift a heavier weight and perform as many repetitions as possible trying to reach 12, or perform more repetitions than 12, always reaching volitional failure. Participants were suggested to lift a heavier weight if they could perform more than 6 repetitions with it or to perform more repetitions if they felt they could not, but to never exceed 18 repetitions. We consider that the fact that participants did not perform the same number of repetitions occasionally did not have any effect on the results, as muscle size improvements are not affected by the number of repetitions performed when the repetition range does not exceed 20 or go under 6 (Baz-Valle et al., 2018; Schoenfeld et al., 2021).

As the duration of each of the phases of an exercise seems to induce regional hypertrophy (Diniz et al., 2020), the eccentric phase of the exercise had to last 2 s, and the concentric phase of the exercise had to be performed as fast as possible for participants in both groups. This was emphasized many times to participants because it is known that a longer time under tension can elicit greater muscle growth (Burd et al., 2012). Participants were also requested to rest at least 48 h between training sessions. Resting between repetitions was not allowed, and participants had to rest from 3 to 5 min between sets. Training sessions took place at gyms in which each participant usually exercised. To test whether participants performed the exercises with the correct technique and with the right intensity, each participant had to send a video performing an effective set to the main researcher after every session. Performing exercises with a bad technique for 3 or more sessions was an exclusion criterion, although none of the participants met it. Participants registered the weight lifted in each set in a mobile phone APP (Dudysolutions version 2.3, 2020 Spain) so that they could see the weight lifted and the repetitions performed in previous sessions, and try perform better.

### Muscle Thickness

**Figure 1:**
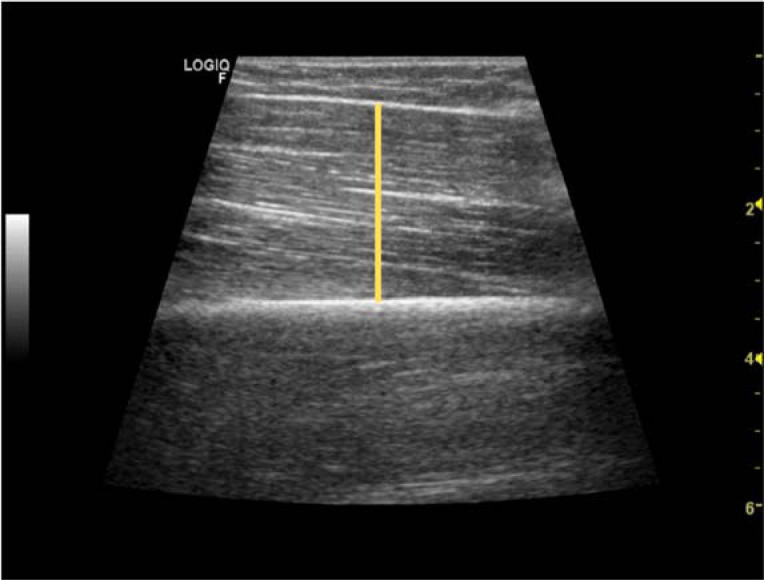
A representative measurement of MT of a subject from an Ultrasound Scan.

Muscle size/thickness was measured using B-mode ultrasound imaging (GE LOGIQTM e, GE Healthcare, WI, USA) with a linear-array transducer (code 12 L-RS, variable frequency band 4.2–13.0 MHz, field of view 3.7 mm) by an experienced technician. Measurements were performed with participants in a supine position, with arms and legs extended and relaxed. Prior to testing, participants remained in this position for 10 min to allow for stabilization of normal body fluids. The technician then applied a water-soluble transmission gel (Aquasonic 100 Ultrasound Transmission gel; Parker Laboratories Inc., Fairfield, NJ, USA) to each measurement site and a 9 MHz ultrasound probe was placed parallel to the tissue interface without depressing the skin. When the quality of the image was deemed as satisfactory, the technician saved the image to the hard drive. Scan acquisition sites were determined by measuring 50%, 60% and 70% of the distance between the posterior crest of the acromion and the olecranon as performed in previous research (Matta et al., 2011). When determined, a circle was drown around the circumference of the arm in those three spots. After that, the center of the biceps brachii muscle was calculated as a line between the coracoid process and the anterocubital crest. Three scans were taken in the place were both lines crossed at 50%, 60% and 70% of the determined length. Muscle thickness (MT) was considered the average of the three measurements. Thickness was measured using the line straight function of ImageJ software (Kim et al., 2020) in the center of the scan (Figure 1). The technician that performed the scans was blinded for the exercises that each participant had performed. Measurements were taken on the right side of the body.

### Anthropometric Measurements

One day before and 3–4 days after the 9-week intervention, anthropometric characteristics of participants were measured. Participants were weighed on a calibrated digital scale whilst wearing minimal clothing. Body height was measured with a stadiometer attached to the scale with participants standing shoeless and head aligned in the horizontal Frankfurt plane. Eight-site skinfold measurements (in mm) were taken from the biceps brachii, triceps, scapular, abdominal, suprailiac, thigh and medial calf sites according to standard procedures using a skinfold caliper (Harpenden1, Baty International, West Sussex, UK). All skinfolds were measured to the nearest 1 mm and the mean of three readings was recorded as the final value for each site. All body composition measurements were taken by the same investigator 24–48 h before and 72–96 h after completion of the training protocol. Body fat percentage (%BF) was estimated using the equation proposed by Faulkner (Faulkner, 1966). It was measured to ensure that participants stayed in a normo-caloric or a caloric-surplus status, and to avoid confounding factors in the ultrasound scans. In some participants, it was not easy to distinguish where the muscle ended and were the skin and subcutaneous fat began. If fat percentage does not increase, any increase in arm thickness cannot be attributed to an increase in fat mass.

### Dietary Guidelines

To avoid the potential for dietary confounding, participants were given a document in which they were instructed to reach 2 g•kg^−1^ of protein intake. First, they were taught how to calculate their protein needs according to their bodyweight. Then, they were instructed on how to reach that amount with examples of different dishes and were requested to include at least 20 g of protein per meal divided into 3 to 5 meals with at least 3 hours between them. Participants were also trained to be in an eucaloric diet or slight energy surplus. Participants also agreed not to take any supplements that could interfere with the study outcomes (such as creatine or whey protein).

### Design and Procedures

**Figure 2:**
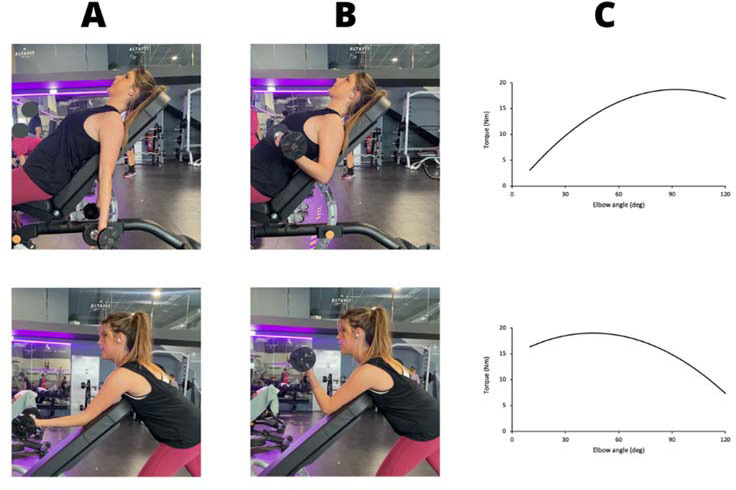
Incline press **(**INC) and preacher curls (PREA) exercises and their resistance profiles (Loss and Candotti, 2008). A. Initial position; B. Final position; C. Resistance torque during elbow flexion.

As the aim of our study was to evaluate the effects of resistance profiles, two exercises with different resistance profiles were chosen for the present study: inclined bicep curls and preacher curls. The first one places the highest strain at the end of the exercise, with the elbow in a 90º–100º position, with the forearm parallel to the floor (Figure 2). As some people usually flex the elbow to a greater degree in this exercise and our aim was to make the end of the concentric phase the hardest part of the RoM, participants were requested not to flex the elbow more than 110º. The resistance increased along the RoM of the exercise, that is, an ascending resistance curve (Figure 2). The second one places the highest srain at the beginning of the exercise, with the elbow in a 0º position (Figure 2). The resistance decreased along the RoM of the exercise, that is, a descending resistance curve. To minimize the influence that the resistance of other exercises may have on the adaptations to the exercises included in the study, participants could not perform any additional exercise that included an active elbow flexion, i.e., neither exercises that directly targeted the bicep such as biceps curls, nor exercises that indirectly trained it such as pull ups. Even if not performing exercises that involved elbow extension was suggested to the participants, they were allowed to do them.

**Figure 3:**
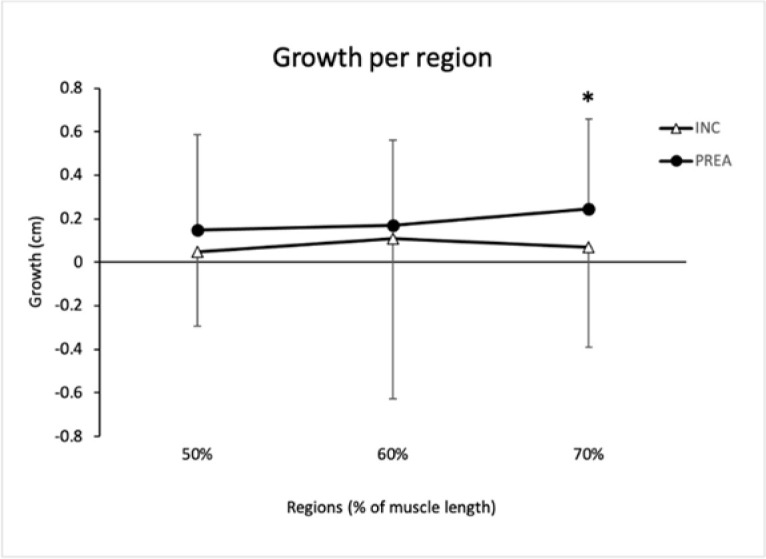
Muscle growth per region (in percentages). * significantly different from pre

To assess muscle growth, MT measurements were performed. MT was chosen instead of the cross sectional area (ACSA) as performing reliable ACSA measurements with an ultrasound apparatus is difficult and operator dependant (Hernández-Belmonte et al., 2022). MT was defined as the thickness of all muscles in the arm (biceps brachii and brachialis) in the field-of-view of the probe, from the skin interface to the humerus. This study was designed to evaluate the effects of two exercises between groups. First, we performed a within-region analysis, to verify whether any of the regions grew in response to the exercise performed. Second, to compare growth between regions, a between-region analysis was performed afterwards.

### Statistical Analysis

We tested all variables for normal distribution (Shapiro-Wilk test) and homogeneity of variance (Levene’s test). An independent sample *t* test was used to check for significant differences in MT and %BF variables prior to the beginning of the study. If the results of the Levene’s test were significant, the Welch’s correction method was used. To analyze MT differences between regions, first, we transformed the increases in MT into percentage increases (%MT). Then a two-way ANOVA was performed with %MT as a dependent variable (Factor 1: group, factor 2: region). When there were significant F values for the main effects or interactions, the Tukey *post-hoc* test was used. To analyze within-region changes in muscle thickness, a paired sample *t* test were applied to compare PRE and POST measurements. In the within-region analysis, the Wilcoxon’s signed rank test was used when the variable did not have a normal distribution. Effect sizes for the ANOVA were calculated as Partial η squared (η_p_^2^). They were considered irrelevant (< 0.01), small (0.01–0.06), medium (0.06–0.14) and large (> 0.14) (Goss-Sampson, 2019). Effect sizes for paired sample *t*-tests were calculated using Cohen’s *d*, and were considered trivial (< 0.2, trivial), small (0.2–0.49 ), moderate (0.5–0.79) and large (≥ 0.8) (Cohen, 1988). An intra-class correlation coefficient test was performed to measure the reliability of the measurement protocol, in a pilot study carried out with ten participants. All the statistical analyses were performed with JASP 0.16 for Mac. The level of significance was set at *p* < 0.05, but was then adjusted using the Benjamin-Hochberg procedure to decrease the False Discovery Rate (FDR < 5%). A pilot study was performed previously to test the reliability of the technician and the ultrasound apparatus. Ten sedentary participants not related to the present study underwent MT measurement protocols in two different days, with at least one week between each measurement. The intraclass correlation coefficient (ICC (3,1)) of the pilot study was calculated using an Excel spreadsheet based on a single rater, absolute agreement and 2-way mixed-effects model, and interpreted as poor (< 0.5), moderate (0.5–0.75), good (0.75–0.9) or excellent (> 0.9) (Koo et al., 2016).

## Results

We found no differences in the body fat percentage (*p* = 0.18) prior to the beginning of the study.

### Between-Group and Region Analysis

The two-way ANOVA showed no interaction for group (*p* = 0.102, η_p_^2^ = 0.032), region (*p* = 0.370, *η_p_^2^* = 0,024) or group*region (*p* = 0.544, η_p_^2^ = 0.015). For this reason, no *post-hoc* analysis was performed.

### Within Regions Analysis

Result of the paired sample *t*-test were not significant in the INC group (*p* = 0.95, *d* = 0.019; *p* = 0.520, *d* = 0.242 and *p* = 0.536, *d* = 0.193 in 50%, 60% and 70% regions, respectively).

The 70% region of the PREA group grew significantly (*p* = 0.017, ES = 0.623), but in contrast, neither the 60% region (*p* = 0.071, *ES* = 0.503) nor the 50% region (*p* = 0.347, *ES* = 0.263) grew.

The intra-class correlation coefficient for the technician and the ultrasound apparatus used for the study was ICC (3,1) = 0.96 (CI = 0.93–0.99), showing excellent reliability between measurements, and demonstrating our protocol to be very highly reliable (Munro, 2005).

**Table 1:** Mean muscle thickness per region in cm (*n* = 32).

	50% PRE	60% PRE	70% PRE	50% POST	60% POST	70% POST
INC	2.19 ± 0.28 [2.04–2.34]	2.3 ± 0.38 [2.09–2.51]	2.74 ± 0.43 [2.51–2.97]	2.24 ± 0.32 [2.06–2.41]	2.41 ± 0.23 [2.28–2.53]	2.81 ± 0.44 [2.57–3.05]
PREA	2.13 ± 0.34 [1.97–2.28]	2.32 ± 0.43 [2.14–2.53]	2.68 ± 0.43 [2.50–2.90]	2.27 ± 0.43 [2.07–2.47]	2.5 ± 0.38 [2.33–2.68]	2.94 ± 0.44 [2.74–3.14]*

INC, inclined biceps curls group; PREA, preacher curls group. Values are Means ± SD [95% CI]

* Significantly different from PRE

**Table 2:** Two-way ANOVA (Group and region) of the %MT.

	Sum of squares	df	Mean square	F	*p*	η_p_^2^
Group	1336.411	1	1336.411	2.740	0.102	0.032
Region	982.326	2	491.163	1.007	0.370	0.024
Group* region	597.508	2	298.754	0.613	0.544	0.015
Residuals	39992.147	82	487.709			

* Significant value

## Discussion

The main finding of this study was that the distal region of the arm grew in response to the preacher curl (an exercise with a descending resistance profile), while the rest of the regions of that group and all the regions of the other group did not. In contrast, between-region comparisons showed no growth differences. This means that while the within-region analysis suggests that the resistance profile of an exercise seems to contribute to the growth of a given region inside the arm muscles, results from the between-region analysis show that there may be many other factors influencing regional hypertrophy as well.

The question arises how resistance profile differences can make some regions grow while the rest does not. We suggest three mechanisms that may provide an explanation for this finding. The first mechanism may be the degree of elongation of the muscle during the exercise. Evidence suggests that training at long muscle lengths may increase sarcomere length (Wisdom et al., 2015), which may elicit further increases in muscle size in the distal region. Some studies report that after performing exercises at long muscle lengths, fascicle length increases to a greater degree than muscle growth (Valamatos et al., 2018), and this may partly explain why changes in MT observed in our study were so small. In this regard, our results are in line with previous studies like the one by Sato et al. (2021) that found a greater increase in MT of the distal region of the arm (70% of the distance between the lateral epicondyle and the acromion) after performing preacher curls at long muscle lengths *vs*. short muscle lengths. This suggests that when the arm muscles work at long muscle lengths, the distal region undergoes higher tension than the rest of the muscle (Nosaka and Sakamoto, 2000).

The second mechanism is related to the coordination of the elbow flexors. The brachialis muscle is more present in the distal regions than in the more proximal ones. In fact, in the scans of some participants that were clear enough to discriminate between the biceps and the brachialis muscles, the thickness of the brachialis at the 70% region was twice as the one found at the 50% region. Probably, working at long muscle lengths stimulates the brachialis muscle more than the biceps brachii muscle. However, due to the length-tension relationship of the elbow flexors, elbow flexion is not very effective at long muscle lengths (Ismail and Ranatunga, 1978). This may have stimulated the brachialis, by far the strongest elbow flexor (Kawakami et al., 1994), to a greater degree than the biceps brachii in the preacher curl exercise.

The third mechanism may be the neural strategy used by each participant to face the requirements of the exercise, as previous research has shown that some people have the ability to selectively choose muscles that perform the same exercise (Avrillon et al., 2021). Recent evidence suggests that the moment generated by each joint can be predicted by decoding the neural signal arriving to the muscles (via HD EMG measurements *in vivo*) (Sartori et al., 2017). Future research on regional hypertrophy should focus on understanding the interplay between the mechanical role of a muscle region and the neural signals it receives, as muscle hypertrophy is a result of both mechanical forces and neural inputs (Alix-Fages et al., 2022).

Regardless of the mechanisms behind the findings of the within-region analysis, it is surprising that no region in the INC group grew, and it is even more surprising that the between-region comparison showed no differences in the regions' growth. However, both groups had a considerable risk of type II statistical error, as 49 participants were needed to reach sufficient statistical power, and neither group reached that number. Thus, the lack of growth may just be a consequence of the small sample size.

In general, the growth observed in the only region is notable (ES = 0.623, medium ES, approximately a 10% increase). One of the reasons why many regions did not experience a significant growth may be that MT was used to assess it. Muscle is a 3-dimensional organ, and as such it can grow in three dimensions. For that reason, the golden standard to measure muscle growth is the Magnetic Resonance Imaging (MRI) (Franchi et al., 2018b). Unfortunately, MRI is difficult to implement and very expensive, and for that reason we chose to measure MT with a b-mode ultrasound apparatus, cheaper and easier to implement. As MT measures only 1D growth, we may have missed part of the growth due to the constraints of our measurement method. Another potential source for the lack of growth in the INC group is the high drop–out rate. Six women dropped out from the INC group, while only one dropped out from the PREA group. Such a drop-out rate increases the probability of a type II statistical error. However, previous research analyzing an exercise with a resistance profile presumably similar to the one used in the INC group (Nunes et al., 2020) (maximal difficulty close to 90º of elbow flexion) found no growth after 8 weeks of training (Drummond et al., 2016). Therefore, another possible explanation for the lack of growth observed in the INC group may be that this kind of resistance does not provide enough stimuli for the growth of elbow flexors. In contrast, a study by Nunes et al. (2020) analyzing the very same exercise found a 7% increase in MT. These contradictory findings imply that even if the resistance profile of an exercise forces the muscle to work at short muscle lengths (which presumably has less potential for hypertrophy), there must be other factors involved.

This study faced several limitations. Muscle is a three dimensional organ, and for that reason the optimum quantification of its growth is three-dimensional (Franchi et al., 2018a). We performed ultrasound scans, which only allows to perform MT or ACSA measurements. Since the techniques used to measure ACSA from ultrasound scans are too complex and operator dependant (Hernández-Belmonte et al., 2022) and MT has a very high correlation with ACSA (Franchi et al., 2018a), we deemed it an acceptable way to measure muscle growth. The ICC shows a high degree of agreement between measurements, that can also be seen elsewhere and confirm that measuring MT with ultrasound scans is a reliable manner to quantify muscle growth (Franchi et al., 2018a; Palmer et al., 2015). However, we could not distinguish whether the measured growth was due to the biceps brachii or brachialis muscle in the scans of many participants, which made it difficult to draw conclusions about the reason of this regional MT increase. A reliability analysis for the skinfold technician was not performed, which may also be a source of potential error, and finally, we must admit that the between-group analysis used in this study may have limited its statistical power. This must be taken into account by researchers aiming to investigate in this field, as a within subject analysis using one arm as control may be more appropriate.

## Conclusions

In conclusion, this study shows that regional hypertrophy is affected by the resistance profile of an exercise. This means that different regions of a muscle will grow in response to exercises that place the highest difficulty in specific points of the range of motion. In particular, our results show that the distal region of the arm grows in response to exercises that place the highest amount of strain in the range of motion in which the arm muscles are more elongated, but not in those that place the greatest strain when the arm flexors are shortened.

According to the results of this study, the resistance profile of any exercise changes the manner in which the involved muscles grow. For example, when aiming to increase the size of the elbow flexors in the part that is closest to the elbow, using exercises in which the highest strain is posed when muscles are elongated seems to be the best option. This is key in sports such as bodybuilding in which shape, size and symmetry of muscles are evaluated in competition.
